# Photonic quasi-crystal terahertz lasers

**DOI:** 10.1038/ncomms6884

**Published:** 2014-12-19

**Authors:** Miriam Serena Vitiello, Michele Nobile, Alberto Ronzani, Alessandro Tredicucci, Fabrizio Castellano, Valerio Talora, Lianhe Li, Edmund H. Linfield, A. Giles Davies

**Affiliations:** 1 NEST, CNR—Istituto Nanoscienze and Scuola Normale Superiore, Piazza San Silvestro 12, 56127 Pisa, Italy; 2 School of Electronic and Electrical Engineering, University of Leeds, Leeds LS2 9JT, UK

## Abstract

Quasi-crystal structures do not present a full spatial periodicity but are nevertheless constructed starting from deterministic generation rules. When made of different dielectric materials, they often possess fascinating optical properties, which lie between those of periodic photonic crystals and those of a random arrangement of scatterers. Indeed, they can support extended band-like states with pseudogaps in the energy spectrum, but lacking translational invariance, they also intrinsically feature a pattern of ‘defects’, which can give rise to critically localized modes confined in space, similar to Anderson modes in random structures. If used as laser resonators, photonic quasi-crystals open up design possibilities that are simply not possible in a conventional periodic photonic crystal. In this letter, we exploit the concept of a 2D photonic quasi crystal in an electrically injected laser; specifically, we pattern the top surface of a terahertz quantum-cascade laser with a Penrose tiling of pentagonal rotational symmetry, reaching 0.1–0.2% wall-plug efficiencies and 65 mW peak output powers with characteristic surface-emitting conical beam profiles, result of the rich quasi-crystal Fourier spectrum.

## Abstract (web summary)

Various vertical surface emitting, terahertz quantum-cascade lasers have been proposed recently but these suffer from power cancellations in the far-field and limited extraction efficiencies. Here, Vitiello *et al.* circumvent these issues using two-dimensional photonic quasi-crystalline resonators.

## Graphical abstract

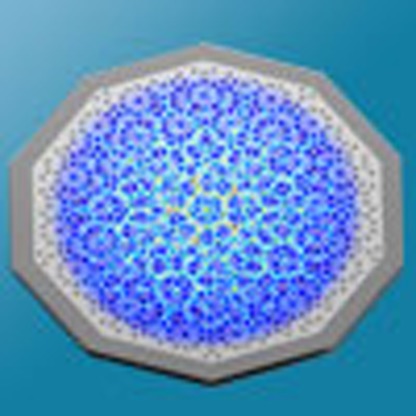

Lasing in two-dimensional (2D) photonic crystals has been the object of extensive recent research^1^,^2^,^3^,^4^,^5^. Operation is normally achieved on modes at the edges of photonic bandgaps or on the localized states formed by suitably designed defects within the periodic photonic lattice. When compared with their periodic counterparts, quasi-crystalline structures^6^,^7^ show significant richness and flexibility in engineering specific device optical properties^8^,^9^, like in random structures^10^. Specifically, the Fourier structure^11^ of quasi-crystalline materials, with or without a photonic bandgap, can be exploited in a laser cavity to engineer the mode frequency and spacing separately, or to control the emission profile independently of the feedback conditions. The first reports of quasi-crystal lasers made use of optically pumped devices, exploiting either a 2D Penrose lattice^12^,^13^ or a resonator with 12-fold symmetry^14^ and operated at visible frequencies. More recently, the 1D photonic quasi-crystal concept has been used with electrically injected THz quantum-cascade lasers (QCLs)^15^, allowing surface emission at chosen angles and wavelengths from tightly-confining double-metal waveguides, although the wall-plug (WP) efficiency was still poor (≈0.01%)^15^.

In general, the use of double-metal waveguides offers considerable advantages for maximizing the operating temperature in THz QCLs, although such devices suffer from the lack of efficient extraction and collimation of the output radiation^16^,^17^. These problems have recently been addressed by engineering 1D edge-emitting third-order distributed feedback lasers (DFBs)^18^, 1D photonic heterostructures^19^,^20^, 2D annular DFBs^21^ and 2D photonic crystal lasers^22^,^23^,^24^. Using such schemes, vertical surface emission with near zero in-plane momentum has been proposed and demonstrated in various implementations, with extraction efficiency values limited by the symmetry of the lasing modes that leads to power cancellations in the far-field. In a periodic structure, indeed, the symmetric and antisymmetric mode feature more than an order of magnitude difference in the quality factor computed including the vertical radiative losses (*Q*_vertical_), thereby meaning that lasing results only on the low radiative-efficiency (conventionally named non-radiative), high quality factor modes.

In this letter, we demonstrate that such power cancellation issues can be elegantly circumvented using quasi-crystalline resonators, in which the distinction between symmetric (vertically radiative, but low quality factor, *Q*) and antisymmetric (non-radiative, high *Q*) modes is fully overcome. In particular, we report the development of high WP efficiency 2D photonic quasi-crystal THz QCLs based on a Penrose P2 (kite and dart) tiling with a five-fold rotational symmetry^6^.

## Results

### Computational model of the quality factor

**Figure 1:**
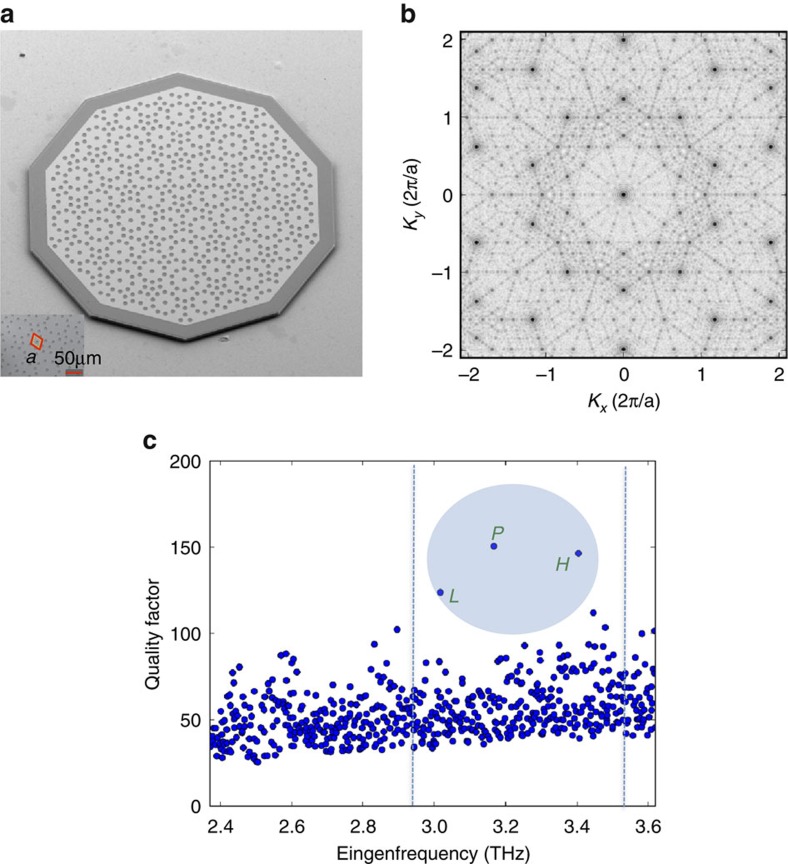
Device image and quality factors. (**a**) Scanning electron microscope image of a prototype device. Holes of radius *r*=6 μm and a lattice spatial length scale *a=*23 μm have been lithographically designed at each vertex of a Penrose pattern and imprinted into the top Cr/Au metallization of the THz QCL (see inset). To implement strong absorbing boundary conditions, a thin (35 μm) Cr (7 nm) border was preliminary evaporated around the mesa pattern. The 75-nm-thick n^+^ contact layer was then removed in the holes to reduce the cavity losses, and decagonal mesa structures were etched down to the bottom metal layer to avoid lateral current spreading. (**b**) Form factor S(**k**) in reciprocal space of the Penrose quasi crystal in **a**. (**c**) Quality factor *Q* of the computed optical modes as a function of the radiation frequency for a device having *r*=6 μm and *a*=23 μm. The dashed vertical lines indicate the QCL gain bandwidth; the circle identifies the optical modes having the highest *Q* factors, labelled with P, L and H.

The QCL active region^25^ is sandwiched between two metallic cladding layers, to create a double-metal waveguide that confines, with an almost unitary confinement factor, the THz radiation in the direction of growth (vertical, *z* axis) and allows its propagation in the *x*–*y* plane, therefore making the device a nearly ideal 2D photonic system. To implement the Penrose crystal, holes were opened in the top metallization of a decagonal mesa structure, at the vertices of the Penrose tiles (see Supplementary Fig. 1 and Supplementary Methods), as shown in the scanning electron microscope image of Fig. 1a. The waveguide mode is strongly modified in the hole regions (as the upper metallic cladding layer is missing locally) and radiation can extend outside the semiconductor. Each opening therefore acts not only as a scatterer for the propagating radiation, but also as an aperture through which radiation can be out-coupled. The ratio *R* between the hole radius *r* and the spatial length scale *a*, is maintained between 0.25 and 0.35 to ensure a sufficient degree of scattering/outcoupling, but without overly increasing the waveguide losses and decreasing confinement^22^. Working with vertical emission, the number of crystal units, chosen here as the smallest one compatible with small mode overlap with the boundaries, controls the lateral confinement of the mode and the ‘probing’ of the absorbing device boundaries and has no influence on the radiative outcoupling.

A full 3D electromagnetic simulation is required to accurately model quasi-crystal resonators. While this is possible, it is extremely demanding in terms of computational resources, given the absence of translational invariance and the presence of thin sub-wavelength metallic layers that require a very fine discretization mesh. As such, we have initially adopted a simplified 2D model^16^, in which we consider the structure as invariant along the *z* axis and represented by an equivalent crystal composed of two materials with different local effective dielectric constants (one for the regions comprising the holes, and one for the unpatterned area). The surrounding region is then modelled as an absorbing layer, leading to smooth boundary conditions for the guided modes (see Supplementary Methods). A full 3D simulation was finally performed to have access to the radiative quality factor (*Q*_vertical_) and to validate the predictions of the 2D model (see Supplementary Methods).

For the transverse magnetic polarization dictated by the selection rules of intersubband transitions, the opening of complete photonic bandgaps, in an array of low refractive index pillars immersed in a high refractive index material, requires much larger filling factors *R* than those adopted here. This is even true for periodic crystals^26^, although it does not preclude lasing at selected **k**-points in the lattice reciprocal space where the local density of photonic states (and hence the net gain) is high^22^. In a band picture, high densities of states are achieved at extremal points where the band dispersion tends to zero and the modes are described by stationary non-propagating waves. In other words, such states are mainly the result of Bragg reflections from the lattice, coupling counter-propagating modes of wavevectors **k** and –**k**, and giving rise to the feedback action necessary for lasing.

Although a band description fails in a quasi crystal owing to the lack of translation invariance, the form factor S(**k**), obtained as a Fourier transform of the spatial profile of the dielectric constant, still contains relevant information on the main Bragg reflection processes. The Fourier spectrum S(**k**) of a quasi crystal is indeed often self-similar, and, in the limit of large sample size, singular continuous^13^, meaning that for every two peaks of S(**k**) there is always a third peak between them. Figure 1b shows the form factor S(**k**) in reciprocal space for the representative Penrose crystal structure shown in Fig. 1a. While a periodic crystal is characterized by a few well-defined reciprocal wavevectors (Bragg peaks), a quasi crystal has a much richer spectrum with many Bragg resonances (ideally, covering the whole reciprocal space)^12^. In our Penrose crystal, which contains about 40 spatial units, several main Bragg peaks are present with a characteristic 10-fold rotational symmetry^12^. Standing waves form in the crystal as a consequence of multiple diffractions on the main reciprocal lattice points, according to the relation


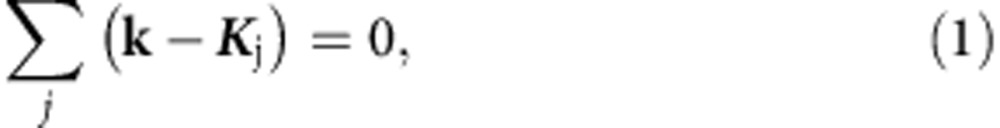


where ***K***_j_ are reciprocal lattice Bragg points and **k** is the optical mode wavevector^13^.

The quality factor *Q* of the computed optical modes, quantifying the energy lost per cycle, is plotted in Fig. 1c as a function of radiation frequency for a prototype device with *R*=0.26 and *a*=23 μm; this reflects the broad spectrum of Bragg resonances apparent in Fig. 1b, and the lack of photonic bandgaps. A few main modes appear with *Q* factors significantly higher than the average, and amongst these we identify three dominant modes labelled P, L and H in Fig. 1c that are well within the gain bandwidth of the QCL^25^. The 2D simulations show that the hole radius and its refractive index both have a strong influence on the *Q* factor and eigenfrequency of the main optical mode P (see Supplementary Fig. 2). It is worth mentioning that the 2D computed *Q*_s_ do not contain the radiative contribution *Q*_vertical_. As a consequence, they mostly give information on the lateral confinement of the optical modes.

**Figure 2:**
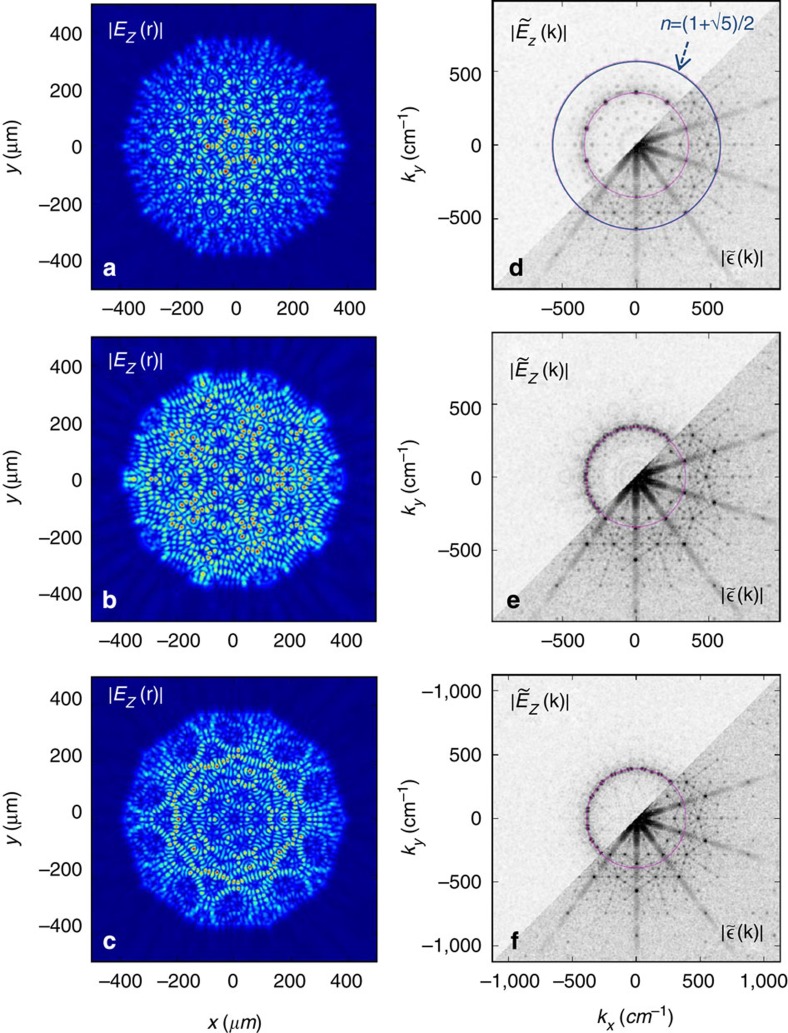
Simulated optical modes. (**a**–**c**) Computed 2D spatial profiles of the electric field modulus for the higher *Q* optical modes of Fig. 1c: (**a**) P, (**b**) L, and (**c**) H. (**d**–**f**) Fourier transform 
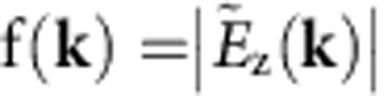
 of the optical mode spatial profiles (upper left) in the reciprocal space for (**d**) P, (**e**) L, and (**f**) H (upper left half), plotted together with the form factor 
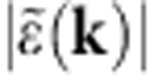
 of the designed quasi crystal (bottom-right half). f(**k**) peaks at values **k**_p_ identified by heavy dots. To identify the Bragg peaks responsible for the feedback, one can overlap the circles of radius nk_*p*_, for each *n* corresponding to values that satisfy the Bragg equation; the circumference with *n*=(1+√5)/2 is shown in blue in (**d**) and perfectly intersects strong resonances of 
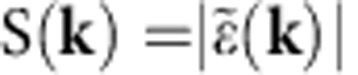
 indicating a five-wave diffraction as the origin of the feedback for the related optical mode P. The circle of radius **k**_p_ is also plotted in pink to show Bragg peaks of S(**k**) responsible for vertical extraction.

Modes with higher *Q* factors can here arise only from a reduced overlap with the outer absorbing boundary, that is, from a spatial distribution mainly localized in the device center; these modes are therefore the principal ones confined by the grating feedback. The calculated 2D spatial profiles of P, L and H, represented by the modulus of the electric field component in the vertical (*z*) direction are shown in Fig. 2a–c, respectively, with their Fourier transforms 
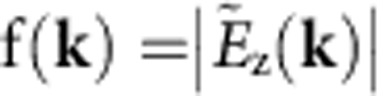
 in reciprocal space shown in Fig. 2d–f (upper left); these present a 10-fold symmetry and have peaks at specific values, **k**_p_. In the bottom-right half of the same panels, the Fourier transform of the spatial profile of the dielectric constant, that is, |ε(**k**)| is also plotted.

To identify the Bragg peaks responsible for the feedback, we can overlap the circumferences of circles corresponding to values of n**k**_p_ that would satisfy eq. (1) for a small finite number of diffractions (for example, *n*=2 for the simplest two-wave coupling **k**_p_−***K***_j_=**−k**_p_, and so on). Their intersections with the relevant points of the reciprocal space, shown in the lower right sections of Fig. 2d–f, indicate which Bragg reflections give rise to the feedback for each mode. The highest-*Q* optical mode, labelled P in Fig. 1c is mainly composed of 10 plane waves, 5 of which are visible in the upper left of Fig. 2d. For this P-mode, the circumference with *n*=(1+√5)/2, shown in blue in Fig. 2d, intersects strong resonances of S(**k**), indicating a five-wave diffraction as the origin of feedback. For the lower frequency (L; Fig. 2b) and higher frequency (H; Fig. 2c) modes, clear intersections with the relevant points in S(**k**) are not found for the main simplest values of *n*, indicating complex multiple diffraction mechanisms^13^.

**Figure 3:**
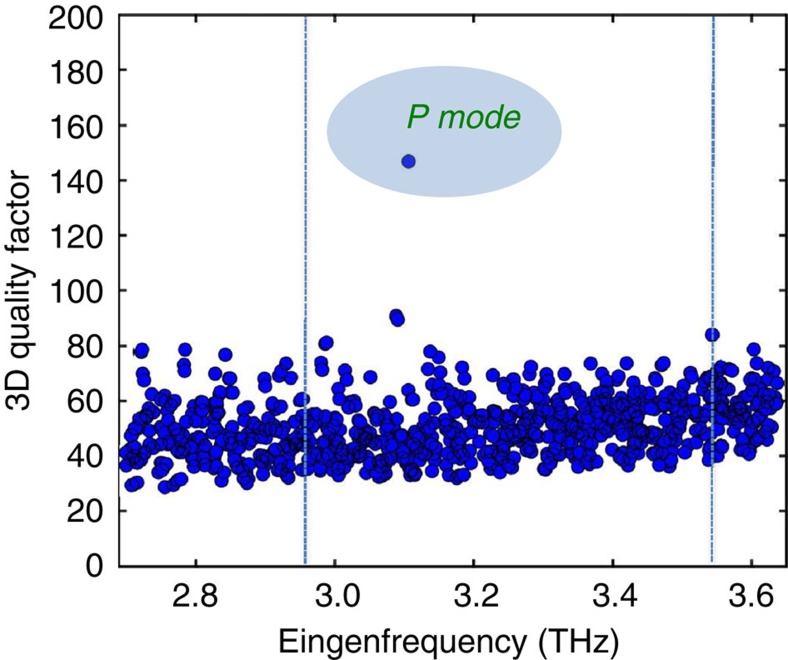
3D quality factors. Quality factor *Q* of the computed optical modes as a function of the radiation frequency for a device having *r*=6 μm and *a*=23 μm. The dashed vertical lines indicate the QCL gain bandwidth; the circle identifies the quality factor of the P-mode.

A full 3D simulation was performed to provide an estimate of the photon loss rate due to surface emission *γ*_r_ (see Supplementary Methods). The vertical radiative losses have been indeed included in the quality factor computation (*Q*_vertical_), therefore allowing to have quantitative information on the emission efficiency. The radiative outcoupling efficiency *η*_r_ is here assumed to be proportional to *Q*_total_/*Q*_vertical_ being *Q*_tot_=(1/*Q*_inplane_+1/*Q*_vertical_)^−1^. The 3D simulations in Fig. 3 show that only one mode, identified with the P-mode, has a total quality factor *Q*_tot_ significantly above the average, while the relative low *Q*_vertical_ of the other main modes in Fig. 1c definitely kills their *Q*_tot_. From the 3D simulation we extract a P-mode total quality factor *Q*_tot_≈147 corresponding to a total photon loss rate *γ*_tot_≈21.1 GHz (see Supplementary Methods). The computed P-mode photon loss rate due to surface emission is *γ*_r_≈3.4 GHz, corresponding to a *Q*_vertical_≈900, and consequently to *η*_r_≈16% and to *Q*_inplane_≈175, meaning that the main resonator photon loss channel is expected to be the non-radiative in-plane one, well supporting the simplified 2D model outcome. It is worth mentioning that there several high frequency modes showing larger *η*_r_ than the P-mode; however, their significantly lower *Q*_tot_ (Fig. 3) makes them unable to lase.

### Terahertz laser emission in quasi-crystal resonators

To realize our devices, the three-well resonant-phonon depopulation THz QCL active region of ref. 25 was employed as the gain medium—this structure has a sufficiently wide gain bandwidth so that the main optical mode can be easily tuned by about 13% from the central frequency; this enables the eigenfrequencies of the predicted high-*Q* modes to be tailored within the gain curve. A set of Penrose-like 2D resonators having a decagonal section were then fabricated by fixing *a*=23 μm and varying *r* in the range 5.8–7.6 μm.

**Figure 4:**
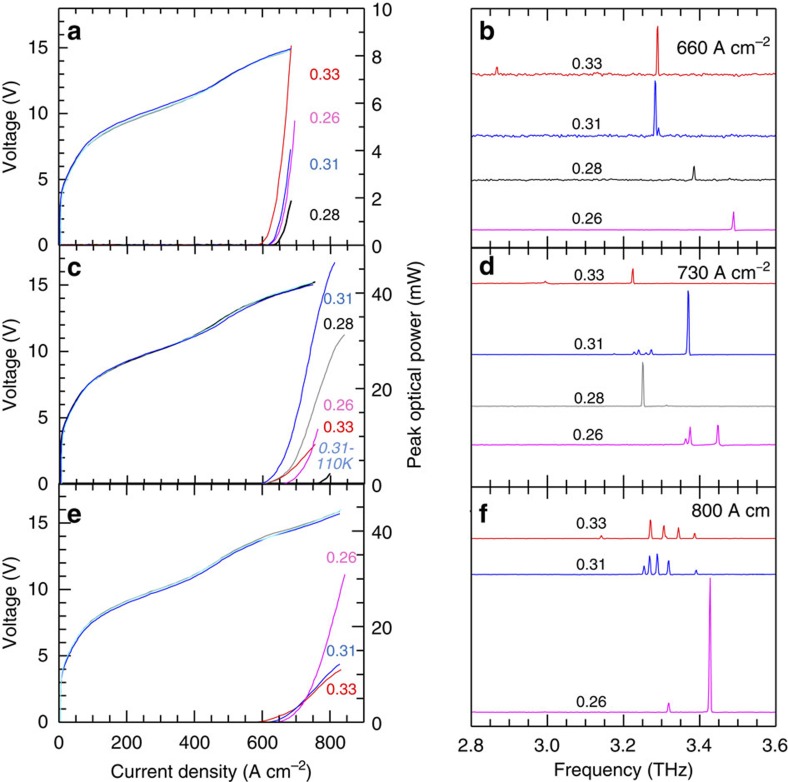
Light–current–voltage and spectral characterization. (**a**,**c**,**e**) Power–current-density (LJ) and voltage–current-density curves as a function of *R=r*/*a* measured at 10 K for different sets of devices designed with a fixed spatial length scale: (**a**) *a*=23, (**c**) 22 and (**e**) 21 μm; the corresponding device areas were about 0.74, 0.67 and 0.60 mm^2^, respectively. Lasers are driven with 1 μs current pulses at a 1% duty-cycle. For the device with a filling factor *R*=0.31 and *a*=22 μm, the corresponding LJ characteristic at a heat sink temperature of 110 K measured with a 2% duty-cycle is also shown. The optical power scales were not adjusted to take into account the 78% transmission coefficient of the thermoplastic cyclic olefin cryostat window and the 90% collection efficiency. (**b**,**d**,**f**) Corresponding laser output spectra for the sets of devices designed with (**b**) *a*=23, (**d**) *a*=22 and (**f**) *a*=21 μm, and different filling factors. The spectra were acquired in rapid scan mode with a resolution of 0.125 cm^−1^ using a Fourier transform interferometer spectrometer and a deuterated triglycine sulfate (DTGS) pyroelectric detector.

Figure 4a shows the measured current density-voltage (J–V) and power—current density (L–J) characteristics for four prototype devices with *a*=23 μm. The threshold current density (*J*_th_) slightly varies from 600 to 640 A cm^−2^ as *R* is changed, with a slope efficiency of 20 mW A^−1^ for the device with *R*=0.33, which has a peak output power of 8 mW at 10 K under pulsed operation. Figure 4b shows the corresponding laser spectra at *J*=670 A cm^−2^. For *R*=0.33, the quasi crystal supports one main lasing mode at 3.29 THz with an additional smaller intensity peak at a lower frequency (2.84 THz). The main peak red shifts by almost 8 GHz when *r* is reduced by 7% (*R*=0.31), a frequency shift smaller than that expected if ones assume the same hole refractive index (≈16 GHz; Supplementary Fig. 2b). In addition, a smaller mode becomes active on the high-energy side. We attribute the main peak for both *R*=0.33 and *R*=0.31 to emission from the dominant P-mode of Fig. 1c and Fig. 3, for which a favourable matching with the peak gain of the active material occurs. However, upon further reducing the hole radius to *R*=0.28 or *R*=0.26, the P-mode (whose frequency should be further red-shifted on the low frequency tail of the gain curve) is no longer visible and a weaker single-mode emission is observed at higher frequencies, associated with the lower-*Q* modes of Fig. 3, for which a more favourable gain condition is now occurring.

It is worth noticing that, as common in a double-metal THz QCL, a large portion (around 500 A cm^−2^) of the threshold injection current is actually due to leakage before the correct band alignment of the structure is reached. As such, threshold current densities only marginally depend on the actual mode *Q* factor and a reliable determination from the experiments is very difficult to be achieved.

To confirm our interpretation, we fabricated another set of samples with the goal of increasing the P-mode frequency by about 5%. Four devices were tailored with *a*=22 μm, and *r* in the range 5.7–7 μm. Figure 4c shows the I–V and L–I characteristics. The peak output power is significantly higher than previously, reaching 45 mW at 10 K for the device with *R*=0.31, suggesting that P is the main mode responsible for laser emission here. The peak power reduces to 3 mW when the *R*=0.31 device reaches a heat sink temperature *T*_H_=110 K (which corresponds to a lattice temperature of about 148 K).^27^ Owing to the large dissipated electrical power, these devices were not forced to operate at higher currents and temperatures to avoid burn-out, therefore hindering a detailed evaluation of the maximum achievable output power and temperatures.

The laser spectra plotted in Fig. 4d for the *a*=22 μm devices are consistent with our interpretation above. Comparing Fig. 4d,b, we observe that: (i) for *R*=0.33, the expected 5% shift of the P-mode pushes it out of the material gain curve while a single-mode emission (related to a lower-*Q* mode) at a lower frequency on the center of the material gain curve is visible; (ii) for *R*=0.31, the emission is quasi single mode with the main P-mode peak blue-shifted by almost 5% with respect to the corresponding peak in Fig. 4b; (iii) for *R*=0.28, the frequency of the P-mode is now shifted towards the center of the material gain curve and the device can now operate on the P-mode, unlike in Fig. 4b. The recorded frequency is ≈120 GHz less than that observed in the *R*=0.31 (*a*=22 μm) sample, a slightly bigger shift than that expected from the 10% radius reduction (which should lead to a 50 GHz frequency drop, see Supplementary Fig. 2b); (iv) for *R*=0.26, the induced frequency shift is still not enough to induce lasing on the P-mode, which remains on the low frequency side of the material gain curve; the device is therefore still lasing on a lower-*Q* mode. A final set of devices was fabricated to induce an additional 5% shift in the main mode frequency while keeping the range of filling factors identical (*a*=21 μm; *r*=5.4–7.0 μm). The LIV characteristics and spectra are plotted in Fig. 4e,f, respectively. A slight variation in *J*_th_ is recorded among the devices with different *R* values. From the spectral analysis we observe that: (i) for *R*=0.33 and *R*=0.31, the P-mode is not visible—this is as expected and in line with previous observation. The P-mode is blue-shifted too far to the high frequency side of the material gain curve, and is not observed; instead, the device emits on a sequence of modes, blue shifted by almost 5% with respect to the corresponding (*R*=0.33; 0.31) devices in Fig. 4d; (ii) for *R*=0.26, emission on the high-efficiency P-mode is now observed, in excellent agreement with the prediction of Fig. 3, once the correct ≈10% scaling of the target frequency is taken into account.

It is worth noting that the optical power scales of Fig. 4a,c,e have not been adjusted to take into account both the 78% transmission coefficient of the cyclic olefin cryostat (COC) window and the estimated 90% collection efficiency. If these were included, maximum peak powers of ≈65 mW are achieved, corresponding to a maximum WP efficiency that approaches 0.1%. This is significantly higher than that obtained from more classical geometries such as edge-emitting or dual-slit DFB THz QCLs based on the same active region/waveguide combination (Supplementary Fig. 3). Such efficiency performance is also larger than that of conventional periodic structures^22^. A simple physical explanation is that in quasi-crystal resonators the optical modes do not have a naturally defined symmetry of the fields in the aperture.

**Figure 5:**
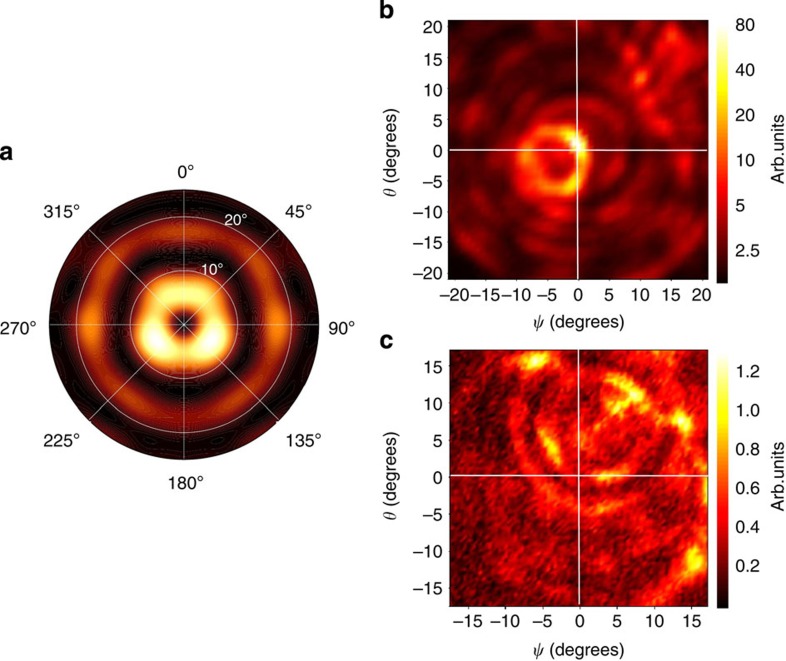
Far-field characterization. (**a**) 3D simulation of the out-of-plane emission of the main optical P-mode of Fig. 1c and Fig. 3, derived by applying the Stratton–Chu method to its near-field emission. (**b**,**c**) Far-field emission patterns of two devices obtained by scanning a pyroelectric detector at a distance of about (**b**) 8 cm and (**c**) 16 cm from the device surface. (**b**) Far-field measured from the device with *a*=21 μm and *R*=0.26. (See Fig. 2f for the emission spectrum of this device.) Similar results have been found for other devices showing P-mode emission (*a*=23 μm, *r*/*a*=0.33 and *r*/*a*=0.31 in Fig. 2b; *a*=22 μm, *r/a*=0.31 and *r/a*=0.28 in Fig. 2d). (**c**) Far-field measured from the device with *a*=21 μm and *r/a=*0.33. (See Fig. 2f for the emission spectrum of this device) Similar results have been found for other devices (*a*=22 μm, *r/a*=0.26 in Fig. 2d; *a*=21 μm, *r/a*=0.31 in Fig. 2f).

The surface emission profiles from these devices can be predicted from S(**k**) and f(**k**). Any Bragg peak **K**_l_ existing within the light cone |**k**−**k**_p_|<2πν/c, centred on **k**_p_, allows out-of-plane scattering, matching the quasi-crystal mode with the free-space radiation of in-plane wavevector **k**_∥_=**k**_p_−**K**_l_, and frequency *ν*. The resulting far-field patterns are then composed of a series of spots at given angles, also featuring a 10-fold symmetry^13^. A special case is that of **k**_p_ being coincident with a given **K**_l_: this scattering gives **k**_||_=0, that is, vertical emission is obtained. This is the case for the P-mode of Fig. 2a,d for which a well collimated emission is expected. A full 3D simulation was performed for this high-*Q* mode, and the far-field pattern was derived from the Stratton-Chu method applied to the near-field emission.^15^ Figure 5a shows the simulation results: the beam is collimated into a 8° ring from the vertical and the 10-fold symmetry is not apparent. This is expected since the spread of each scattering emission is point-like only in the limit of an infinite quasi crystal and of a fully spatially coherent optical mode^13^. In the present case, the modes occupy only a few tens of spatial crystal units, meaning that the preferred angles of emission broaden into more homogeneous ring-like shapes. Figure 5b shows the related experimental measurement. The emission of the quasi crystal (*R*=0.26, Fig. 4e,f) is well collimated into a ≈10° ring from the vertical with further weaker rings at larger angles, in qualitative agreement with the simulation. Such a ring profile can be converted in a more regular single-lobed pattern by introducing a defect in the central area of the pattern or by grading the hole size across the quasi-crystal pattern, that is,. by breaking the resonator rotational symmetry^23^. For sake of comparison Fig. 5c, shows the angular emission extrapolated for a different sample in which no P-mode emission was detected (*R*=0.33 in Fig. 4e,f). The emission is here spread in a concentric and more chaotic sequence of rings covering a wider 15° angle, in agreement with the predicted multiple diffraction mechanisms at the origin of the feedback.

## Discussion

To make our conclusions more general, we applied the same resonator concept to a different THz QCL active region design (diagonal resonant-photon structure)^28^, showing a significantly smaller (0.1 THz) gain bandwidth and larger internal quantum efficiency at 3.8 THz. A 0.2% maximum WP efficiency with peak powers of 65 mW has been reached here with similar far-field profiles compatible with P-mode emission. Such efficiency performance is competitive with the best surface-emitting 1D or 2D THz QCLs resonators (see Supplementary Fig. 4).

In conclusion, we have shown that a 2D photonic quasi-crystal concept can be exploited to realize efficient injection lasers based on a quantum-cascade active medium in the far-infrared (THz) region of the spectrum. By fabricating a Penrose tiling with pentagonal rotational symmetry in the QCL top metallization layer, highly efficient vertical emission with peak output powers of ≈65 mW and WP in the 0.1–0.2% range can been achieved. Such powers/efficiencies are larger than those achieved with conventional^22^ or uniformly graded 2D photonic crystal lasers^23^ at very low temperatures (4 K), in corresponding double-metal devices, showing the potential of quasi-crystalline structures for the development of more efficient photonic devices and micro-cavity lasers, as well as for practical metrological and spectroscopy applications across the far-infrared. Furthermore, the described approach may be employed in other physical problems where symmetries need to be broken yet preserving the general order of the system.

## Methods

### Fabrication procedure

The QCL (sample L341) was grown by molecular beam epitaxy on an undoped GaAs substrate and consists of a GaAs/Al_0.15_Ga_0.85_As heterostructure based on the design reported in ref. 25. The growth sequence started with a 250 nm undoped GaAs buffer layer, and was followed by a 300 nm Al_0.5_Ga_0.5_As etch-stop layer, a 75 nm layer of GaAs n-doped to 5 × 10^18^ cm^−3^, 226 repetitions of the gain medium creating a 10-μm thickness active region, and a final 50 nm GaAs layer n-doped to 5 × 10^18^ cm^−3^. The thicknesses (in nm) of the active region layers are: **4.8**/9.6/**2.0**/7.4/**4.2**/16.1, with a 5.6 nm thick portion of the underlined GaAs well being doped to *n*=5 × 10^16^ cm^−3^ and the Al_0.15_Ga_0.85_As barriers being depicted in bold face. After growth, the QCL wafer was thermo-compressively bonded with an Au–Au interface on an n^+^-GaAs carrier wafer. After selective removal of the host GaAs substrate by etching, and removing the Al_0.5_Ga_0.5_As etch-stop layer, the active region was coated with a top Cr/Au (5 nm/150 nm) metallization. Holes of radius *r* between 5.4–7.7 μm, on a lattice spatial length scale *a*=23–21 μm, were then lithographically patterned in the metal at each vertex of the Penrose pattern. The 75-nm n^+^ contact layer was removed in the holes by reactive-ion etching process to reduce the cavity losses. To implement strong absorbing boundary conditions the pattern was surrounded by a pre-defined thin Cr (7 nm) frame extending 35 μm around the Penrose pattern. This Cr border acted as a mask during the reactive-ion etching process, preventing the n^+^ top contact layer from being etched away at the periphery of the Penrose pattern where the absorbing boundary is required. As a final processing step, decagonal mesa structures were etched down to the bottom metal using a H_2_SO_4_:H_2_O_2_:H_2_O (11:9:50) etching solution to avoid lateral current spreading. Individual devices were indium soldered onto a copper block and symmetrically wire-bonded around the decagon border to ensure uniform current injection through the mesa, while avoiding any perturbative effects in the far-field.

### Optical characterization

The lasers were mounted on the cold finger of a helium-flow cryostat, and were driven with 1 μs current pulses at a 1% duty-cycle. For the spectral characterization, laser emission was collected with a f/1 parabolic mirror, passed through a Michelson Fourier transform interferometer, and measured in rapid scan with a deuterated triglycine sulfate pyroelectric detector. The light–current curves were measured with a pyroelectric detector at a distance of ≈2.5 cm from the device, without any collection optics and without applying any correction for the cryostat window transmission.

The far-field data were acquired with a pyroelectric detector, which had a sensitive area of about 7 mm^2^ and was mounted at a fixed position. The device was scanned on a *x*–*y* translation stage, driven by stepper motors with a spatial resolution of ≈0.2 μm, in the plane perpendicular to the growth direction, at distances of 8 cm and 16 cm from the sample.

## Author contributions

M.S.V., A.T and A.R. devised the concept; A.R., F.C. and V.T. performed the simulations; M.N. fabricated the devices; M.S.V. and M.N. performed the experiments; L.L., E.H.L. and A.G.D. carried out the growth of QCL materials; M.S.V. analysed the data; M.S.V. wrote the manuscript with the contributions from the other authors. M.S.V. and A.T. coordinated the whole project.

## Additional information

**How to cite this article:** Vitiello, M. S. *et al.* Photonic Quasi-crystal Terahertz Lasers. *Nat. Commun.* 5:5884 doi: 10.1038/ncomms6884 (2014).

## Supplementary Material

Supplementary Information Supplementary Figures 1-4, Supplementary Methods and Supplementary References
