# Supplementary material for: Photonic quasi-crystal terahertz lasers
Source: Nat Commun. 2014 Dec 19;5:5884. doi: 10.1038/ncomms6884 (PMC4284629; doi:10.1038/ncomms6884)
Supplement: Supplementary Information — Supplementary Figures 1-4, Supplementary Methods and Supplementary References [file ncomms6884-s1.pdf]

Supplementary Information

Supplementary Figures

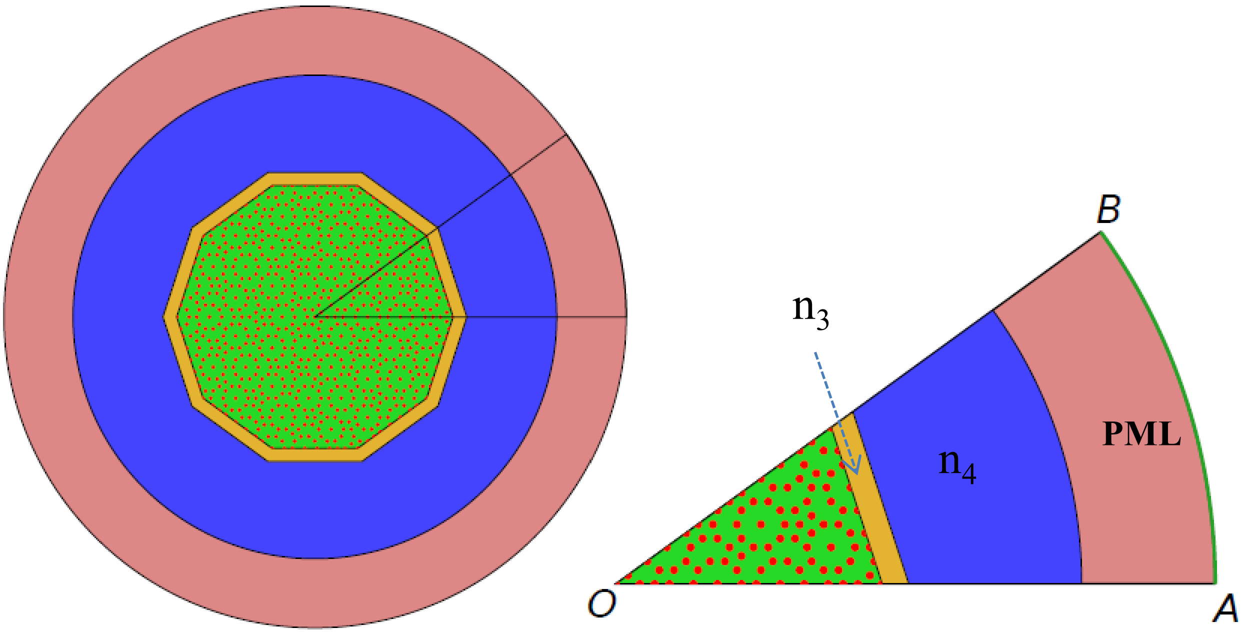

Supplementary Figure 1. Simulated device geometry. Simulated decagonal mesa geometry with the main dielectric area having refractive index n_1 = 3.6, filled by circular scatters having (refractive index n_2). A surrounding region with a 35 μm width, and a complex effective index n_3=3.6+i was used to model the absorbing layer, defining smooth boundary conditions for the guided modes. An external region, having n_4 = 1.0 and terminated by perfectly matching layer (PML) conditions, was used to model the open boundaries outside the mesa device. The radius of the circle inside which the decagon is inscribed is 400 μm.

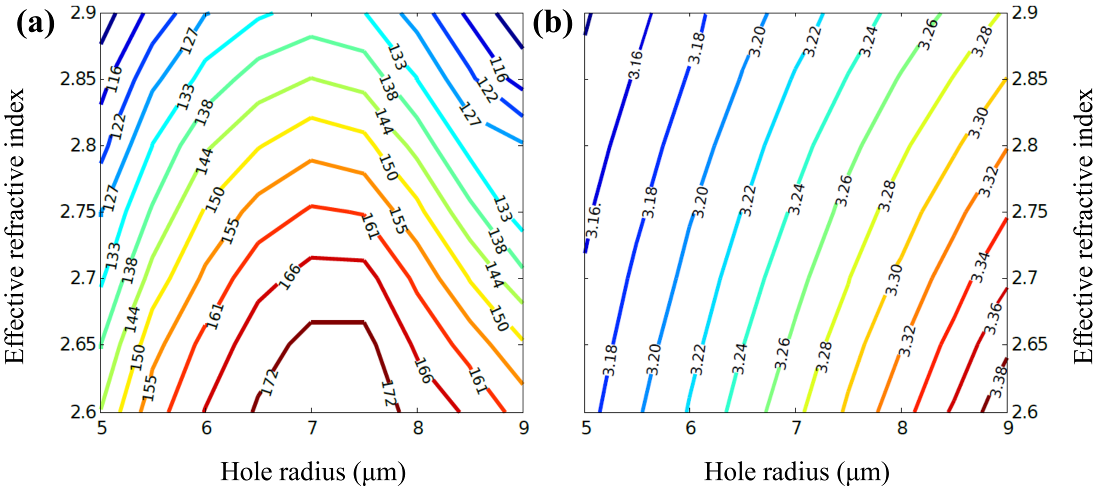

Supplementary Figure 2. Mapping of the main optical mode. (a,b) Maps of the Q factor (a) and resonant frequency (in THz) (b) of the highest Q optical mode as a function of the hole radius r, and refractive index n_2.

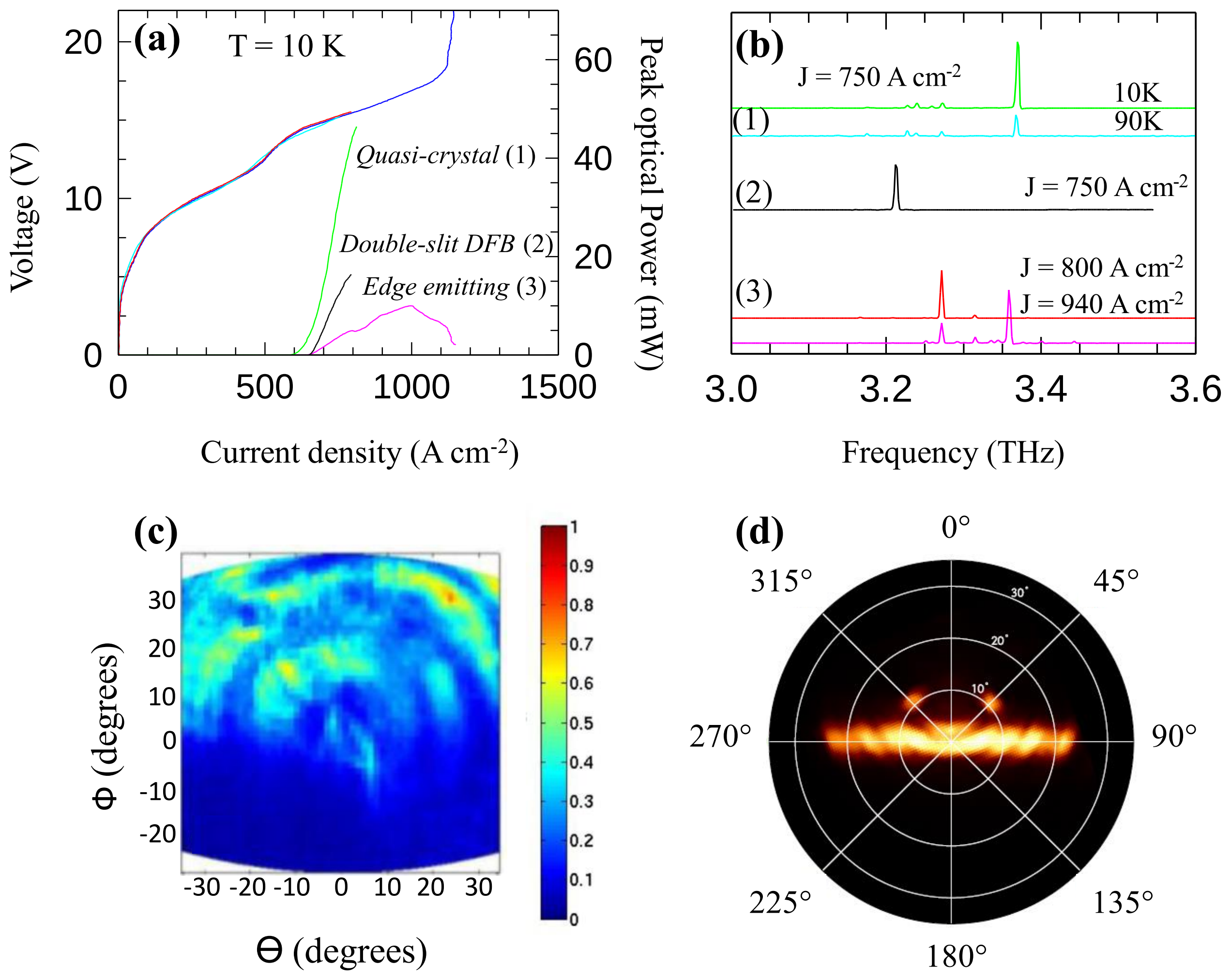

Supplementary Figure 3. Light–current–voltage and spectral characterization. (a) Power–current-density (LJ) and voltage–current-density curves measured at 10 K for: a double metal THz QCL 220 μm wide and 2.1 mm long; a dual slit DFB THz QCL 220 μm wide and 2.2 mm long; a Penrose quasi-crystal designed with spatial length scale: (a) a = 22 μm and a device area of about 0.67 mm^2. Lasers are driven with 1μs current pulses at a 1% duty-cycle. The optical power scales were not adjusted to take into account the 78% transmission coefficient of the thermoplastic cyclic olefin (COC) cryostat window. **(b)** The corresponding laser output spectra. The spectra were acquired in rapid scan mode with a resolution of 0.125 cm^{-1} using a FTIR spectrometer and a DTGS detector. **(c,d)** Far-field emission patterns of the edge emitting (c) and the dual slit DFB (d) device obtained by scanning a pyroelectric detector at a distance of about 10 cm from the device surface, without any collecting optics in the middle. Zero angles refer to the direction orthogonal to the device facet (c) and to the device surface (d), for the edge emitting and the DFB device, respectively.

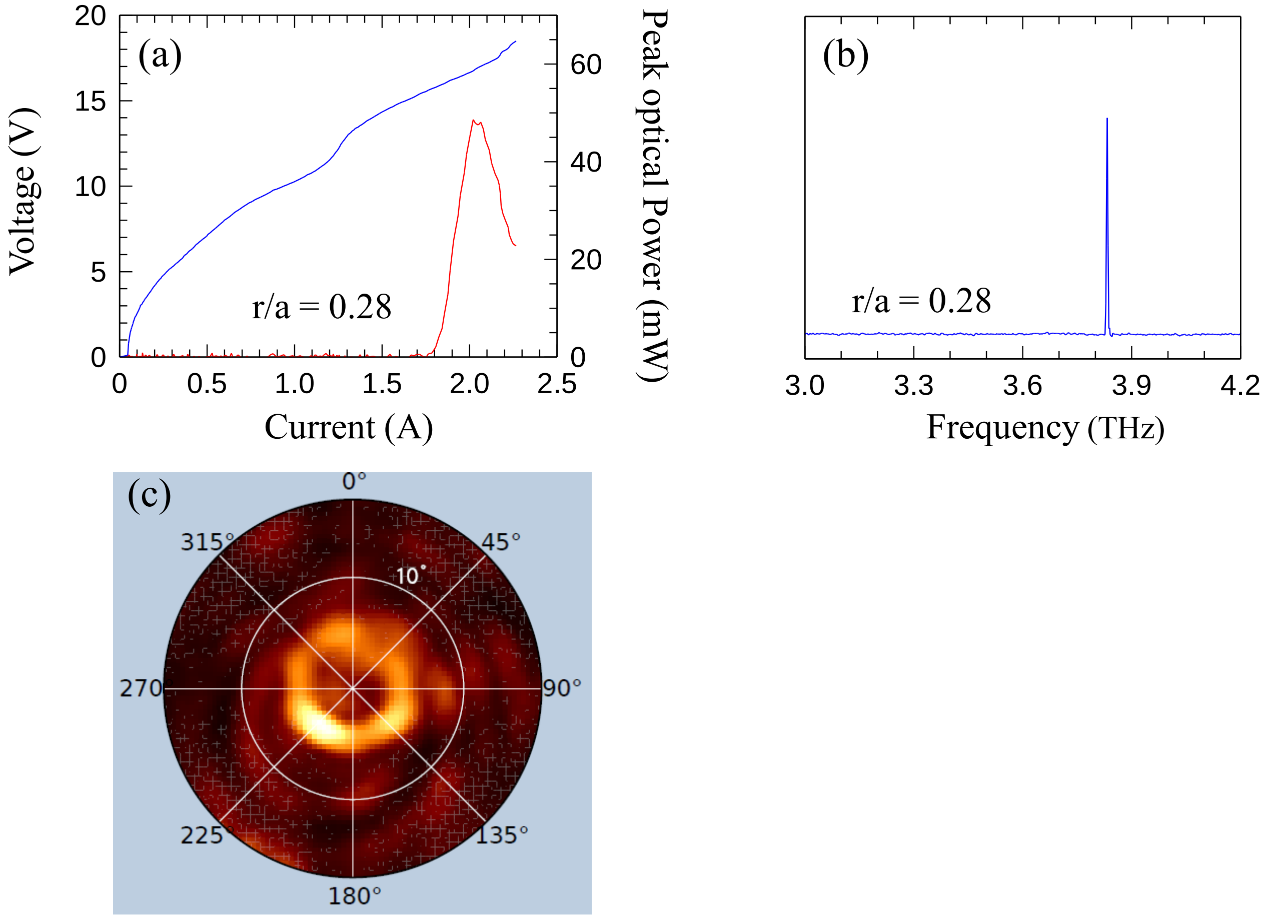

Supplementary Figure 4. Light–current–voltage and spectral characterization. (a) Power–current (LI) and voltage–current curves measured at 10 K for a Penrose quasi-crystal designed with spatial length scale a = 22 μm and having r = 6.16 μm . Lasers are driven with 1μs current pulses at a 1% duty-cycle. The optical power scales were not adjusted to take into account the 78% transmission coefficient of the thermoplastic cyclic olefin (COC) cryostat window and the 90% collection efficiency. (**b)** Corresponding laser output spectra acquired in rapid scan mode with a resolution of 0.125 cm^{-1} using a FTIR spectrometer and a DTGS detector. (**c)** Far-field emission patterns obtained by scanning a pyroelectric detector at a distance of about 8 cm from the device surface.

Supplementary Methods

Simulations. A commercial finite element (FEM) solver was used to compute the eigenvalue solutions to Maxwell's equations for our 2D system (illustrated schematically in Supplementary Fig. 1). Effective dielectric constants were calculated by solving the Helmholtz equation for an infinite slab waveguide with and without top metallization, giving effective refractive indices $n_1 = 3.6$, and $n_2 = 2.7$ in the metallized and non-metallized regions, respectively. This approximation may seem rather crude for a patterned device, as the fields are no longer uniform in the vertical direction due to the presence of the apertures.[1] Indeed, it is known that it induces deviations in the calculation of the eigenfrequencies; nonetheless, it gives a good qualitative representation of the existing modes and their symmetries, and allows the far-field emission to be calculated successfully, assuming that the magnetic field of the eigenmode in the non-metallized areas is the same as the near-field.[2] A surrounding domain, having a complex effective index $n_3 = 3.6 + i$, was used to model the absorbing layer, providing smooth boundary conditions for the guided modes. This is required[2] to ensure that the effective dielectric contrast is the leading confinement mechanism. In the fabricated devices, the high losses represented by the imaginary part of the effective index in this domain were obtained by deliberately preventing the top n^+ contact layer from being removed by the RIE step. Finally, an external domain having $n_4 = 1.0$ and terminated by perfectly matching layer (PML) conditions was used to model the open boundaries outside the mesa.

Resonating eigenfrequencies, quality factors (Q*),* and TM spatial profiles for the photonic modes were numerically obtained from the eigenvectors and complex eigenvalues of the FEM model. By varying the radius r of the patterned holes, we estimated its influence on both the Q factor and the eigenfrequency of the main optical modes. Supplementary Figs. 2a and 2b illustrate the results obtained when studying the highest Q mode, P*,* for a prototype device having filling factor R = r/a = 0.26 and spatial length a = 23 μm. At fixed n_2, the computed eigenfrequency blue shifts by almost 80-100 GHz when r is increased from 5 μm to 7.3 μm (our experimental range),

and the related Q factor progressively rises until r reaches ≈7 μm. Above this value an additional optical mode starts to compete with the main one, making the assignment of the principal modes more difficult. Furthermore, by keeping r fixed, a 10% reduction of the hole refractive index (n_2) can be seen to induce an almost linear blue shift of the computed frequency up to 40 GHz, meaning that the expected optical mode frequency could be affected by such a perturbation.

A full 3D simulation was finally performed to assess the total quality factor, the out-of-plane emission properties of the photonic resonator, both in terms of the effects on the complex eigenfrequency of the confined modes as well as on the angular dependence of the far field emission. A minimal 3D model of the device was obtained by considering the top and bottom metallization as perfect electric conductor (PEC) planar boundary conditions. The QCL heterostructure was approximated by an average refractive index n_l=3.5 under the patterned waveguide and n_3 for the outer absorbing domain, similar to the 2D numerical calculations. The 3D simulation cell was completed by an outer domain with refractive index n_4, terminated by boundaries transparent to in-plane cylindrical waves and out-of-plane planar waves. The FEM solver provides the electromagnetic eigenmodes for this model, along with their complex eigenfrequencies.

The 3D simulations provide an estimate of the photon loss rate due to surface emission γ_r. This has been estimated by extracting from the 3D FEM simulation the time-averaged integrated power flow Φ through the open air domains and normalizing it with respect to the resonator energy E_{res}:

$$\gamma_r = \frac{\Phi}{E_{res}} = \frac{\int_A (E \times H) \cdot \hat{n}\, dS}{\int_V (\epsilon |E|^2 + |H|^2/\mu)\, dV}$$

where E and H represent the electric and magnetic fields respectively, ϵ the dielectric constant and μ the permeability. The value $\gamma_r \simeq 3.4$ GHz has been achieved. The corresponding quality factors have been derived from the relation Q = ν/γ_r, where ν is the P-mode eigenfrequency. It is worth mentioning that a 3% deviation in the mode frequency has been found among the 2D and 3D

simulations, as expected[1] and well within the deviations induced by the perturbation of n_2 and r (Supplementary Fig. 2).

The near field over the waveguide was used to compute the far field emission pattern using the Stratton-Chu method.[1]

Fabrication and experimental details. To make a systematic comparison of our quasi-crystal devices with more conventional cavity geometries, we fabricated an edge emitting double-metal QCL and a double-slit surface emitting DFB both from the same wafer (L341).

After selective removal of the host GaAs substrate by etching, the $Al_{0.5}Ga_{0.5}As$ etch-stop layer was removed. In the case of the standard edge emitting QCL, ridge waveguides were etched down to the bottom metal using a H_2SO_4:H_2O_2:H_2O (11:9:50) etching solution. The active region was then coated with a top Cr/Au (5 nm/150 nm) metallization and back coated. For the DFB structure, a pair of 2-µm-wide slits, of separation d, were lithographically patterned into the top Cr/Au metallization at a fixed period Λ = 26 µm, approximately equal to the wavelength of the mode propagating in the waveguide, while keeping $d/\Lambda = 0.6$. The 75 nm n+ contact layer was removed in the holes by reactive-ion etching (RIE) to reduce the cavity losses. In order to implement strong absorbing boundary conditions, the pattern was surrounded by a pre-defined thin Cr (7 nm) frame extending 20 µm away from each side of the grating pattern. As a final processing step, ridge structures were etched down to the bottom metal using a H_2SO_4:H_2O_2:H_2O (11:9:50) etching solution to avoid lateral current spreading.

Individual devices were indium soldered onto a copper block and wire-bonded at both ends of the longitudinal section to ensure uniform current injection through the device, whilst avoiding any perturbative effects in the far-field.

A threshold current density of 660 A cm^{-2} was measured at 10 K with a central laser frequency of 3.25 THz (Supplementary Ref. 3), and a maximum peak output power of 10 mW in a standard edge emitting double metal geometry (Supplementary Fig. 3). Supplementary Fig. 3a shows the

comparison between the quasi-crystal THz QCL of Fig. 3d (main text) (R = 0.31), a standard double-metal QCL, and the dual slit THz QCL. Wall plug efficiencies of 0.01% and 0.027% were achieved, respectively, for the latter two devices, worse than that of the corresponding quasi-crystal device (0.08%). Owing to the larger dissipated electrical power, the dual slit THz QCL and the quasi-crystal THz QCLs were not forced to operate at higher currents in order to avoid burn-out, therefore hindering a detailed evaluation of the maximum achievable output power.

Supplementary Fig. 3b shows the corresponding laser spectra. While multimode emission is observed in the edge emitting laser, the DFB radiates on the targeted 3.22 THz frequency. It is worth noticing that, as expected, the operating temperature does not induce any significant change in the laser frequency ($\Delta\nu \approx 3$ GHz shift) of the P mode in the quasi-crystal THz QCL.

Supplementary Figs. 3c and 3d show the far field profiles of the double-metal and dual slit THz DFB QCLs, respectively. The high divergent double-metal QCL beam profile is converted into a more regular elongated emission vertically distributed on a 25° angle.

Supplementary Figs. 4a and 4b show the measured current-voltage (I–V) and power-current (L–I) characteristics and the corresponding laser spectra, of a prototype Penrose resonator device with a = 22 µm and r = 6.16 µm based on the active region design of Ref. 4. Once including the 78% transmission of the cryostat window and the 90% collection efficiency corrections, a 0.2% maximum wall-plug efficiency with a peak power of 65 mW has been reached at 3.83 THz. The related far-field profile (Supplementary Fig. 4c) is still compatible with the P-mode emission and comparable to that observed in previous L341 samples showing a single mode emission ascribed to the P-mode. This wall-plug efficiency is competitive with the best surface-emitting 1D DFB THz QCL resonators and only slightly below the typical values obtained in single plasmon THz QCLs (0.3%).

Supplementary References

[1] Mahler, L. & Tredicucci, A. Photonic engineering of surface-emitting terahertz quantum cascade lasers. *Laser Photon. Rev*. **5**, 647-658 (2011).

[2] Chassagneux, Y. *et al.* Electrically pumped photonic-crystal terahertz lasers controlled by boundary conditions. *Nature* 457, 174-178 (2009).

[3] Belkin, M.A. *et al.* Terahertz quantum cascade lasers with copper metal-metal waveguides operating up to 178 K. *Optics Express* 13, 3242-3248 (2008).

[4] Kumar, S., Hu, Q. & Reno, J.L., 186 K operation of terahertz quantum-cascade lasers based on a diagonal design. *Appl. Phys. Lett* .**94**, 131105 (2009)
